# Tanshinone IIA is superior to paricalcitol in ameliorating tubulointerstitial fibrosis through regulation of VDR/Wnt/β-catenin pathway in rats with diabetic nephropathy

**DOI:** 10.1007/s00210-023-02853-3

**Published:** 2023-11-22

**Authors:** Jing-Yi Zeng, Yu Wang, Fu-Yuan Hong, Miao Miao, Yu-Ying Jiang, Zi-Xuan Qiao, Yun-Tao Wang, Xiao-Rong Bao

**Affiliations:** 1 Department of Nephrology, Fujian Provincial Hospital, Shengli Clinical Medical College of Fujian Medical University, Fuzhou, China; 2 Department of Nephrology, Jinshan Hospital of Fudan University, Shanghai, China

**Keywords:** Tanshinone IIA, Paricalcitol, Diabetic nephropathy, Tubulointerstitial fibrosis, VDR/Wnt/β-catenin pathway

## Abstract

Glomerulosclerosis and tubulointerstitial fibrosis (TIF) are closely involved in the development of diabetic nephropathy (DN). Moreover, the development of TIF is closely related to epithelial-to-mesenchymal transition (EMT). Tanshinone IIA (Tan) has various pharmacological effects, especially the anti-fibrotic effect. And it is mainly used in the clinical treatment of cardiovascular diseases. Currently, the protective effect of Tan on DN and its possible mechanism have not been clearly elucidated. Our previous studies illustrated that Tan could improve the EMT of HK-2 cells induced by high glucose by regulating the vitamin D receptor (VDR)/Wnt/β-catenin pathway. Here, we collected demographic information and laboratory results from the National Health and Nutrition Examination Survey (NHANES) database in order to investigate the relationship between VD and DN. Then, we established a DN model and treated DN rats with Tan and paricalcitol (Par) for 6 weeks. We subsequently compared the changes in general condition, renal function, pathological changes, and TIF-related protein expression levels of control rats, DN rats induced by STZ, DN rats with Tan at 5.4 mg/kg, DN rats with Tan at 10.8 mg/kg, and DN rats with Par at 0.054 µg/kg, to explore the effect and mechanism of Tan and Par on DN rats. The results showed that VD had a protective effect against DN in diabetic patients. And we found that Tan had a protective effect on renal fibrosis in DN rats, which was superior to Par in improving the symptoms of “three more and one less,” reducing fasting blood glucose level, improving renal index, BUN/SCr, and UACR, reducing histopathological damage of kidney, and improving the expression of fibrosis-related proteins in kidney tissue by regulating VDR/Wnt/β-catenin pathway. Tan was superior to Par in ameliorating tubulointerstitial fibrosis by regulating VDR/Wnt/β-catenin pathway in rats with diabetic nephropathy.

**Supplementary Information:**

The online version contains supplementary material available at 10.1007/s00210-023-02853-3.

## Introduction

The prevalence of diabetes is increasing year by year, and it is estimated that 536.6 million adults worldwide have diabetes (Sun et al. 2022). Diabetic nephropathy (DN) caused by diabetic microangiopathy is characterized by glomerulosclerosis and tubulointerstitial fibrosis (TIF) when it has progressed to end-stage renal disease (ESRD). Moreover, the development of TIF is closely related to epithelial-to-mesenchymal transition (EMT). TIF is progressive and irreversible, which is a major hindrance to treatment. Given the increasing prevalence and poor prognosis of DN (Tuttle et al. 2022a), it is critical to find effective therapeutic targets.

Tanshinone IIA (Tan), one of the effective components of Salvia miltiorrhiza Bge., has been reported to play an important role in anti-inflammation, anti-oxidative stress, dilation of vascular endothelium, inhibition of cell differentiation by regulating PI3K/AKT/mTOR, JNK, NF-κB, and other signal pathways (Zhang et al. 2022a; Peng et al. 2022; Chen et al. 2022). In recent years, cardiovascular disease is the main cause of death of patients with DN. Tan is currently mainly used in clinical treatment of cardiovascular diseases (Guo et al. 2020), including myocardial infarction (Lu et al. 2021), heart failure (Zhang et al. 2022b), acute ischemic stroke (Ji et al. 2017), and pulmonary hypertension (Wang et al. 2022). More importantly, Tan has been shown to have anti-fibrotic effects in other animal models. For example, Tan could ameliorate bleomycin-induced idiopathic pulmonary fibrosis (Xue et al. 2021). Previously, we reported that Tan could attenuate high glucose-induced EMT in HK-2 cells by regulating vitamin D receptor (VDR)/Wnt/β-catenin (Zeng and Bao 2021). However, the effects and mechanisms of Tan on DN remain incompletely unclear. To explore the effects of Tan on DN rats, and to provide a theoretical basis for the clinical application of Tan in the treatment of DN, we established a DN model and administered Tan at different concentrations and paricalcitol (Par).

## Materials and methods

### Study population

**Fig. 1:**
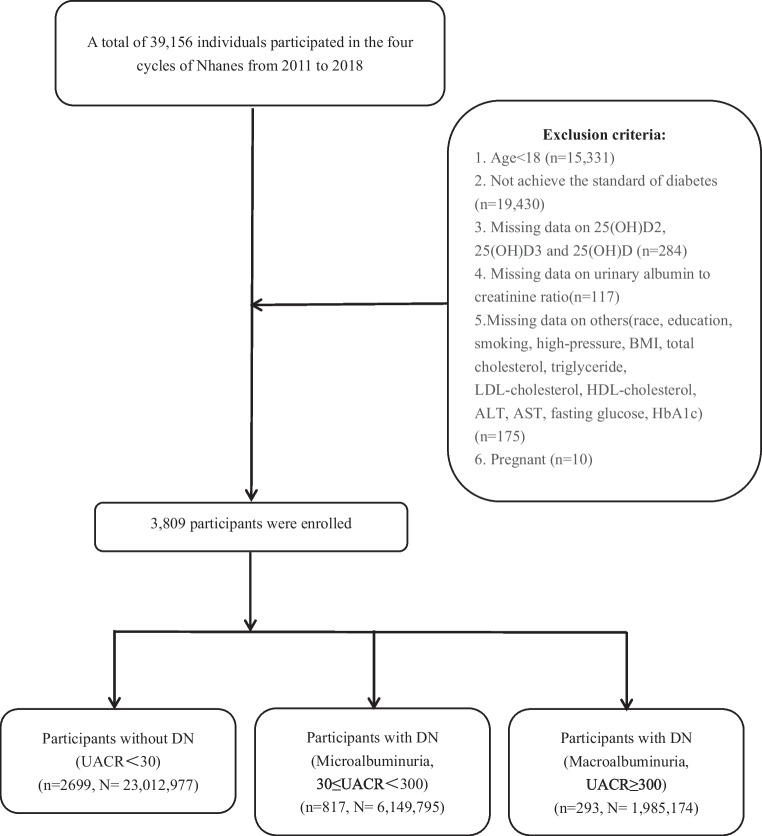
Flowchart of patient selection in this study

The National Health and Nutrition Examination Survey (NHANES) database, including a nationally representative sample’s information on demographic, socioeconomic, dietary, and health-related questions, is designed to assess the health and nutritional status of Americans. As shown in Fig. 1, the participants’ data was extracted from NHANES from 2011 to 2018, and a total of 39,156 participants were initially included. The exclusion criteria were as follows: (1) under 18 years old; (2) not meet diagnostic criteria for diabetes (the definition of diabetes: (a) answering “Yes” to the question “Doctor told you have diabetes?” in the NHANES questionnaire module; (b) taking insulin; (c) taking diabetic pills to lower blood sugar; (d) glycohemoglobin (HbA1c) ≥ 6.5%; (e) fasting blood glucose (FBG) ≥ 7.0 mmol/L; (f) oral glucose tolerance test (OGTT) ≥ 11.1 mmol/L); (3) missing important data on 25-hydroxyvitamin D (25(OH)D), 25(OH)D2, 25(OH)D3, urine albumin to creatinine ratio (UACR), and other relevant covariates such as race, education, and smoking; (4) pregnant woman. Then, 3809 individuals were included in our study. Based on the UACR level, participants were categorized into the following three groups: non-DN group (UACR < 30 mg/g), microalbuminuria group (30 mg/g ≤ UACR < 300 mg/g), and macroalbuminuria group (UACR ≥ 300 mg/g).

### Laboratory measurement of serums 25(OH)D2, 25(OH)D3, and 25(OH)D

All serum samples were processed, stored at − 30 ℃, and shipped to the Division of Laboratory Sciences, National Center for Environmental Health, Centers for Disease Control and Prevention, Atlanta, GA, for analysis. Although 25(OH)D is not the bioactive 1,25-(OH)D form, it is the predominant circulating form of vitamin D and is therefore considered to be the most reliable indicator of vitamin D status. The 25(OH)D2 and 25(OH)D3 levels were measured utilizing ultra-high performance liquid chromatography-tandem mass spectrometry (UHPLC-MS/MS, Thermo Fisher Scientific, MA, USA). Then, these are summed to total 25(OH)D.

### Covariates

General characteristics (sex, age, race, and education level), the body mass index (BMI), smoking status, and data on hypertension were collected through home interview, and biochemical tests (total cholesterol (TC), triglyceride (TG), LDL-cholesterol, HDL-cholesterol, aspartate aminotransferase (AST), alanine aminotransferase (ALT), uric acid (UA), FBG, HbA1c, blood urea nitrogen (BUN), serum creatinine (SCr), UACR) were measured in laboratory. More details of the above-mentioned indicators were accessible to the public on the NHANES website.

The race was categorized as Mexican American, other Hispanic, non-Hispanic white, non-Hispanic black, and others. The education level was classified as less than high school, high school or equivalent, and college or above. The BMI was divided into three classes: ≤ 24.9, 25 to < 30, ≥ 30 kg/m^2^. The smoking data was classified as never smoker (smoked < 100 cigarettes in life), former smoker (smoked ≥ 100 cigarettes but quit smoking), and current smoker (smoked ≥ 100 cigarettes and still smoked). Individuals with physician-diagnosed hypertension, or currently taking antihypertensive medication, or with systolic blood pressure ≥ 140 mmHg or diastolic blood pressure ≥ 90 mmHg were identified as hypertensive.

### Statistical analysis of NHANES

Statistical analysis of NHANES was conducted using R version 4.3.1. Referring to the NHANES analytic guidelines, weighted data were used in all analyses of NHANES. Data are expressed as the median (interquartile range, IQR) for continuous variables and percentages (%) for categorical variables. The Wilcoxon rank-sum test was used for continuous variables, and the chi-squared test with Rao and Scott’s second-order correction was applied for categorical variables. The logistic regression models were used to explore the association of 25(OH)D with the risk of DN. A value of *P* < 0.05 was considered to be statistically significant.

### Materials

Tanshinone IIA injection (H31022558, > 96% (HPLC)) was purchased from No. 1 Biochemical & Pharmaceutical (Shanghai, China). Paricalcitol injection (H20183043) was purchased from Hengrui Biotechnology (Jiangsu, China). Streptozotocin (STZ) (S0130) and pentobarbital sodium salt (P3761) were purchased from Sigma-Aldrich (St. Louis, MO, USA). α-SMA Rabbit Monoclonal Antibody (#19,245), GSK-3β Rabbit Monoclonal Antibody (#12,456), β-catenin Rabbit Monoclonal Antibody (#8480), and E-cadherin Mouse Monoclonal Antibody (#14,472) were purchased from Cell Signaling Technology (CST, MA, USA). E-cadherin Mouse Monoclonal Antibody (#ab231303) was purchased from Abcam (MA, USA). VDR Rabbit Monoclonal Antibody (#ET1704-09) was purchased from Huabio (Zhejiang, China). GAPDH Mouse Monoclonal Antibody (#M20006F) was presented by Abmart (Shanghai, China). All other materials were obtained from standard sources.

### Animals

A total of 50 male Sprague–Dawley (SD) rats, weighing 220 ± 20 g at 6–8 weeks of age, were purchased from Shanghai Jiesijie Laboratory Animal Co., Ltd. (Shanghai, China; license number: SCXK(Hu)2018–0004) and kept in an SPF-grade animal laboratory in the Shanghai Public Health Animal Laboratory Building (Shanghai, China). The temperature in the animal laboratory was maintained at 22 ℃ ± 2 ℃ and humidity at 50% ± 10%, with a regular light–dark cycle (12 h/day, light on at 8:00 a.m.). Every 3–4 rats were housed in cages lined with wood chips for bedding, and the bedding was changed every 7 days before modeling and every day after modeling. Except for fasting, all rats had free access to adequate food and water throughout the experiment. All animal experimental procedures were in accordance with the Guide for Institutional Animal Care and Use and approved by the Shanghai Public Health Clinical Center Laboratory Animal Welfare and Ethics Committee (2021-A057-01).

### Preparation of drug

#### Preparation of 0.1 M citric acid-sodium citrate buffer

Citric acid-sodium citrate buffer was composed of citric acid (2.1 g) and sodium citrate (2.94 g). The powder of citric acid and sodium citrate was diluted by 50 mL ddH_2_0 respectively and mixed in a ratio of 1:1.32. The pH value of the mixture was adjusted to 4.2–4.5 using pH test paper. The mixture was filtered with a 0.22-µm filter and then stored at 4 ℃.

#### Preparation of 1% STZ

We weighed STZ powder (STZ was stored in a 50-mL centrifuge tube covered with tin foil and protected from light during the entire process, and STZ dose was calculated according to the fasting body weight of rats) and dissolved 1 g of STZ in 100 mL of 0.1 M citric acid-sodium citrate buffer pre-cooled at 4 °C. The powder was vortexed until dissolved and then quickly placed in an ice-water mixture. The prepared 1% STZ solution was to be used within half an hour.

### Model establishment and drug administration

#### Model establishment

After 7 days of acclimatization, the blood glucose levels of rats were detected by blood collected from the tail vein, and 0 rats with abnormal random blood glucose were excluded. The rats (*n* = 50) were randomly divided into the control group (*n* = 10) and the model group (*n* = 40). After fasting for 14 h, the rats in the model group were given intraperitoneal injections of 1% STZ at a dose of 60 mg/kg, while the rats in the control group were given intraperitoneal injections of the same dose of citric acid-sodium citrate buffer. To prevent hypoglycemia caused by the destruction of islet β cells and the massive release of insulin, the rats in the model group ingested 6% glucose solution orally within 2 h after the STZ injection, and all rats were given standard chow and water ad libitum. After 72 h of the STZ injection, the random blood glucose of the rats was detected. If the blood glucose was no less than 16.7 mM for three consecutive times and maintained for 7 days, the model of diabetes was considered to be established successfully. Forty rats in the model group were successfully modeled.

#### Drug administration

**Fig. 2:**
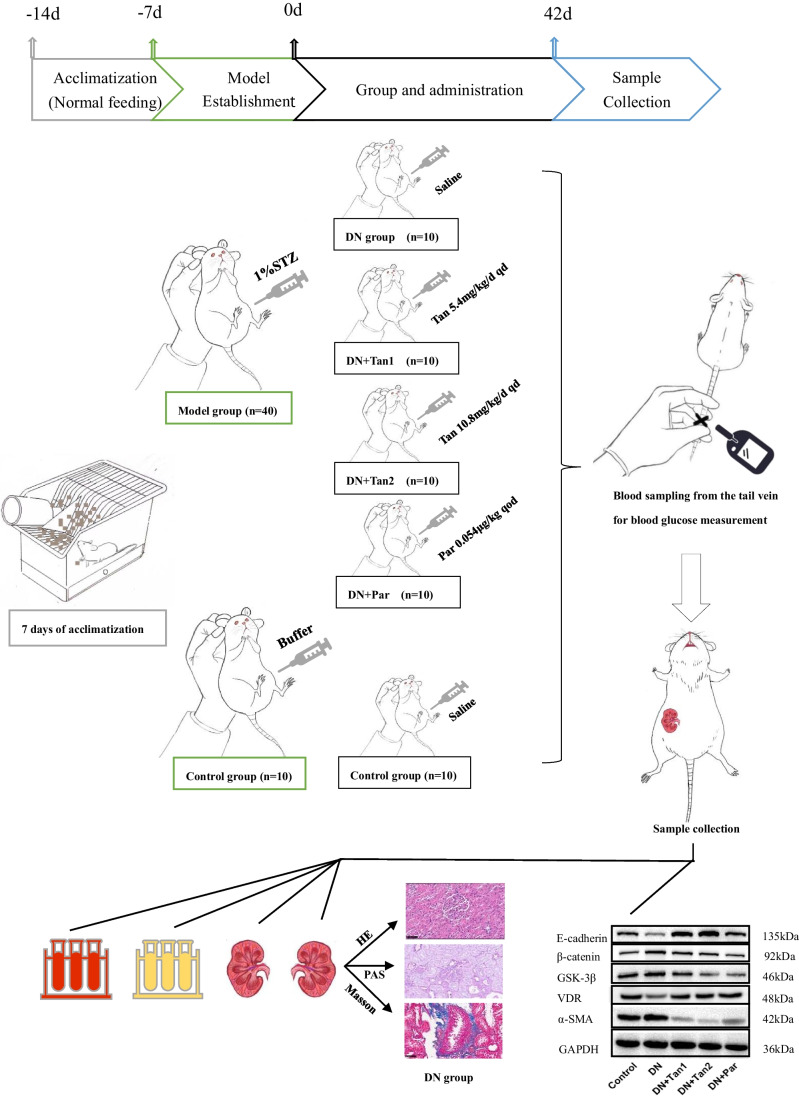
Flow chart. After 7 days of adaptive feeding, the model was established, and the drug was administered. And then samples were collected for testing 42 days after administration. We found that Tanshinone IIA is superior to paricalcitol in ameliorating tubulointerstitial fibrosis in DN rats, which can improve the symptoms of “three more and one less,” reduce FBG level, improve renal index and UACR, reduce histopathological damage of kidney, and improve the expression of fibrosis-related proteins in kidney tissue by regulating VDR/Wnt/β-catenin pathway

The rats (*n* = 50) were randomly divided into five groups (*n* = 10/group): the control group (0.9% saline 1 mL/kg, intraperitoneal injection), the DN group (0.9% saline, intraperitoneal injection), the DN + Tan1 group (Tan 5.4 mg/kg, intraperitoneal injection), DN + Tan2 group (Tan 10.8 mg/kg, intraperitoneal injection), the DN + Par group (Par 0.054 µg/kg, intraperitoneal injection). Saline and Tan were administrated once a day for 42 days, while Par was administrated once every 2 days for 42 days (Fig. 2).

##### Conversion of drug dosage between humans and rats

We regard human weight as 60 kg and rat weight as 200 g, and the drug dosage of the rat is 5.4 times of human clinical drug dosage after equivalent conversion according to the unit weight. Therefore, the dosage of the DN + Tan1 group and the DN + Par group is the equivalent dosage after conversion, and the dosage of the DN + Tan2 group is twice that of the DN + Tan1 group.

### Daily monitoring

The rats’ general condition was recorded throughout the experiment, including body weight, FBG, hair greasiness, eye state (cataracts or other fundus lesions), behavioral activities, and moisture and odor of bedding.

### Sample collection

After administration for 42 days, the 6-h fasting rats were anesthetized by intraperitoneal injection of 5 mL/kg 1% pentobarbital sodium. The blood sample was collected by cardiac puncture, centrifuged at 3000 rpm/min for 15 min at 4 ℃, and then the serum was obtained and stored at − 80 ℃. Similarly, the urine sample was collected by bladder puncture, centrifuged at 3000 rpm/min for 15 min at 4 ℃, and the supernatant was obtained and stored at − 80 ℃ for further testing. After collecting the blood and urine, both kidneys were taken. The kidney was cleaned with 0.9% saline, and the left kidney was immersed in 4% paraformaldehyde (the volume of paraformaldehyde is 15 times the volume of the left kidney) and fixed for 24 h for subsequent pathological staining. In the meanwhile, the right kidney was quickly frozen in liquid nitrogen and stored at − 80 ℃ for subsequent molecular biological detection (Fig. 2).

### Blood and urine examination

Blood samples were tested for serum creatinine (SCr), urea, calcium, and phosphorus using a serum automatic biochemical analyzer (Siemens AG, Munich, Germany). Urine samples were collected to detect urine albumin and creatinine, and UACR was calculated.

### Histopathological analysis

After fixation for 24 h, the left kidney tissue was paraffin-embedded and sectioned. Next, the 5 µm paraffin sections were stained with hematoxylin and eosin (H&E), periodic acid-Schiff (PAS), and Masson’s trichrome. The stained sections were observed under a light microscope (Olympus, Tokyo, Japan) and analyzed using Image-J (NIH, Bethesda, MD, USA).

#### Tubular injury score

According to the irregular arrangement of renal tubules, detachment of brush border, and inflammatory cell infiltration, a semi-quantitative analysis was performed to evaluate the degree of tubular damage. Ten non-overlapping visual fields of each H&E staining Sect. (400 ×) were evaluated and scored (from 1 to 5) independently by two blinded pathologists. (The proportion of damaged renal tubules in the whole renal tubules: 1 (< 11%), 2 (11–25%), 3 (25–50%), 4 (50–75%), 5 (75–100%).) The average of 10 visual fields was taken for analysis.

#### Paller score

To more clearly demonstrate the effect of the drug, further Paller score was performed on PAS-staining sections when the tubular injury scores of the five groups were statistically significant. Ten non-overlapping visual fields of each PAS staining Sect. (400 ×) were randomly selected, and ten renal tubules in each field were evaluated and scored independently by two blinded pathologists. The scoring criteria are shown in Table 1. The maximum score for each renal tubule was 6, and the maximum score for each visual field was 60. The average of ten non-overlapping fields was taken for analysis.

**Table 1:** The scoring criteria of the Paller score

Score	Tubular dilation and flat cells	Brush border	Vacuolar degeneration	Necrotic cells in the lumen of the renal tubules
0	No tubular dilation and flat cells	Intact brush border	No vacuolar degeneration	No necrotic cells in the lumen of the renal tubules
1	Evident tubular dilation and flat cells	Damage of brush border	Vacuolar degeneration	Some necrotic cells in the lumen of the renal tubules but no cell fragments or tube-type cells
2	/	Detachment of brush border	/	Tube-type cells in renal tubules

#### Inflammatory cell infiltration

Ten non-overlapping visual fields of each H&E staining Sect. (400 ×) were evaluated and scored independently by two blinded pathologists. The number of inflammatory cells was counted, including plasma cells, lymphocytes, neutrophils, eosinophils, and monocytes. The average of ten non-overlapping fields was taken for analysis.

#### Relative area of collagen

In Masson’s trichrome staining, collagen deposition is shown in blue, and the ratio of the blue area to the visual field area is the relative area of collagen. Ten non-overlapping visual fields of each Masson’s trichrome staining Sect. (400 ×) were analyzed by Image-J to quantify the distribution and degree of collagen. The average of 10 visual fields was taken for analysis.

### Western blotting

The total protein was extracted from the right kidney tissue according to previous methods (Zeng and Bao 2021), and the concentration of the total protein was measured. Next, 20 µg of proteins were concentrated and then separated by 10% SDS-PAGE and transferred to PVDF membranes. Finally, the membranes were blocked in a quick-blocking buffer at room temperature for 15 min, followed by the incubation of the following primary antibodies (1:1000) at 4 ℃ overnight: α-SMA Rabbit mAb (#19,245), GSK-3β Rabbit mAb (#1245), β-catenin Rabbit mAb (#8480), E-cadherin Mouse mAb (#ab231303), VDR Rabbit mAb (#ET1704-09), and GAPDH Mouse mAb (#M20006). After washing with TBST 3 times, the membranes were incubated with corresponding secondary antibodies (1:5000) at room temperature for 1 h. The expression level of proteins was quantified by Image-J.

### Statistical analysis of animal experiment

Statistical analysis of animal experiment was performed using GraphPad Prism 8.0.2 (GraphPad Software, San Diego, CA, USA). All data were expressed as the mean ± standard deviation (SD). Whether the statistics meet the normal distribution was tested using the Shapiro–Wilk test, and then statistically significant differences were estimated using ordinary one-way ANOVA with Tukey’s test. A value of *P* < 0.05 was considered to be statistically significant.

## Results

### Participants characteristics

There were 817 individuals with microalbuminuria and 293 individuals with macroalbuminuria, representing 6,149,795 and 1,985,174 residents of the USA, respectively. Table 2 presents the characteristics of diabetic patients according to UACR levels. Compared with diabetic patients without DN, patients with higher levels of UACR tended to be older, lower educational attainment, current smoker, hypertension, and tended to have higher levels of TC, TG, UA, FBG, HbA1c, BUN, SCr, and lower levels of HDL, ALT, 25(OH)D3, and 25(OH)D.

**Table 2:** Characteristics of the study participants with diabetes according to UACR levels

Variables	Non-DN group (UACR < 30) (*n* = 2699, *N* = 23,012,977)	Microalbuminuria group (30 ≤ UACR < 300) (*n* = 817, *N* = 6,149,795)	Macroalbuminuria group (UACR ≥ 300) (*n* = 293, *N* = 1,985,174)	*P*-value
Sex				0.3
Male	12,000,940 (52.15%)	3,133,816 (50.96%)	1,180,319 (59.46%)	
Female	11,012,037 (47.85%)	3,015,979 (49.04%)	804,855 (40.54%)	
Age	60.00 (50.00, 68.00)	63.40 (52.19, 73.00)	60.00 (52.00, 74.00)	< 0.001
Race				0.2
Mexican American	2,207,382 (9.59%)	692,554 (11.26%)	224,045 (11.29%)	
Other Hispanic	1,414,713 (6.15%)	397,777 (6.47%)	136,796 (6.89%)	
Non-Hispanic White	14,119,839 (61.36%)	3,529,450 (57.39%)	1,029,982 (51.88%)	
Non-Hispanic Black	3,076,905 (13.37%)	856,565 (13.93%)	365,739 (18.42%)	
Others	2,194,138 (9.53%)	673,449 (10.95%)	228,612 (11.52%)	
Education level				0.021
Less than high school	4,465,412 (19.40%)	1,463,401 (23.80%)	529,154.05 (26.66%)	
High school or equivalent (including GED)	5,702,093 (24.78%)	1,659,257 (26.98%)	463,262.11 (23.34%)	
College or above	12,845,472 (55.82%)	3,027,137 (49.22%)	992,758.00 (50.01%)	
Smoking status				0.023
Never smoker	12,043,827 (52.33%)	2,794,346 (45.44%)	843,597 (42.49%)	
Former smoker	7,548,269 (32.80%)	2,439,282 (39.66%)	757,115 (38.14%)	
Current smoker	3,420,881 (14.87%)	916,167 (14.90%)	384,462 (19.37%)	
BMI				0.058
≤ 24.9	2,411,057 (10.48%)	766,973 (12.47%)	313,619 (15.80%)	
25–29.9	6,574,803 (28.57%)	1,489,911 (24.23%)	451,417 (22.74%)	
> 30	14,027,117 (60.95%)	3,892,911 (63.30%)	1,220,138 (61.46%)	
Hypertension				< 0.001
No	7,618,172 (33.10%)	1,338,808 (21.77%)	189,849 (9.56%)	
Yes	15,394,805 (66.90%)	4,810,987 (78.23%)	1,795,325 (90.44%)	
TC (mmol/L)	4.60 (3.88, 5.38)	4.63 (3.90, 5.51)	4.76 (3.98, 6.10)	0.008
TG (mmol/L)	1.76 (1.20, 2.57)	1.91 (1.29, 2.76)	2.01 (1.32, 3.33)	0.001
LDL (mmol/L)	2.43 (1.81, 3.12)	2.34 (1.80, 3.06)	2.37 (1.78, 3.18)	0.8
HDL (mmol/L)	1.16 (0.98, 1.42)	1.16 (0.96, 1.40)	1.09 (0.85, 1.32)	0.007
ALT (U/L)	23.00 (17.00, 31.00)	21.00 (16.00, 31.00)	21.00 (16.00, 28.00)	0.016
AST (U/L)	22.00 (18.00, 29.00)	22.00 (18.00, 29.00)	21.00 (17.00, 27.00)	0.3
UA (µmol/L)	327.10 (273.60, 386.60)	333.10 (274.43, 410.40)	381.33 (303.30, 434.20)	< 0.001
FBG (mmol/L)	6.83 (5.72, 8.83)	7.44 (6.05, 10.71)	8.31 (6.33, 12.90)	< 0.001
HBA1c (%)	6.60 (6.00, 7.50)	7.00 (6.30, 8.50)	7.40 (6.53, 9.10)	< 0.001
BUN (mmol/L)	5.00 (3.93, 6.43)	5.71 (4.28, 7.14)	7.14 (4.64, 10.00)	< 0.001
SCr (µmol/L)	76.02 (63.65, 90.17)	77.79 (63.55, 98.12)	102.54 (72.78, 144.13)	< 0.001
UACR (mg/g)	8.44 (5.43, 12.99)	61.53 (41.86, 116.55)	781.36 (458.86, 1,542.78)	< 0.001
25(OH)D3 (nmol/L)	65.33 (44.80, 83.80)	59.87 (40.30, 82.00)	48.44 (33.31, 75.82)	< 0.001
25(OH)D2 (nmol/L)	1.45 (1.45, 1.45)	1.45 (1.45, 2.12)	1.45 (1.45, 1.45)	0.3
25(OH)D (nmol/L)	70.90 (51.20, 89.24)	66.43 (45.35, 88.64)	55.99 (38.90, 84.87)	0.001

Data are expressed as weighted number (weighted percentage) for categorical variables and median (interquartile range) for continuous variables

*n*, unweighted number; *N*, weighted number; *BMI*, body mass index; *TC*, total cholesterol; *TG*, triglyceride; *LDL*, LDL-cholesterol; *HDL*, HDL-cholesterol; *ALT*, alanine aminotransferase; *AST*, aspartate aminotransferase; *UA*, uric acid; *FBG*, fasting blood glucose; *HbA1c*, glycohemoglobin; *BUN*, blood urea nitrogen; *SCr*, serum creatinine; *UACR*, urine albumin to creatinine ratio

Then, according to the levels of serum 25(OH)D, the participants were categorized as Q1 (< 25.0 nmol/L), Q2 (25 to < 50 nmol/L), Q3 (50 to < 75 nmol/L), and Q4 (≥ 75 nmol/L). As shown in Table 3, the prevalence of microalbuminuria and macroalbuminuria increased significantly in diabetic patients as 25(OH)D levels declined. Participants with higher 25(OH)D levels were more likely to have lower levels of TC, TG, LDL, HDL, ALT, AST, FBG, HbA1c, and UACR. In contrast, participants with higher 25(OH)D levels had higher levels of BUN and SCr than those with lower 25(OH)D levels. However, BUN and SCr remained in the normal range.

**Table 3:** Characteristics of the study participants with diabetes according to serum 25(OH)D levels

Variables	Q1 (*n* = 180, *N* = 1,019,650)	Q2 (*n* = 979, *N* = 6,951,920)	Q3 (*n* = 1231, *N* = 9,771,137)	Q4 (*n* = 1419, *N* = 13,405,238)	*P*-value
Sex					0.003
Male	443,425 (43.49%)	3,478,692 (50.04%)	5,697,579 (58.31%)	6,695,380 (49.95%)	
Female	576,225 (56.51%)	3,473,228 (49.96%)	4,073,558 (41.69%)	6,709,859 (50.05%)	
Age	52.00 (44.34, 61.00)	56.00 (45.00, 64.00)	59.00 (49.00, 67.00)	64.00 (55.00, 74.00)	< 0.001
Race					< 0.001
Mexican American	225,585 (22.12%)	1,062,980 (15.29%)	1,195,003 (12.23%)	640,413 (4.78%)	
Other Hispanic	60,819 (5.96%)	573,386 (8.25%)	833,402 (8.53%)	481,680 (3.59%)	
Non-Hispanic White	199,272 (19.54%)	3,069,026 (44.15%)	5,601,275 (57.32%)	9,809,698 (73.18%)	
Non-Hispanic Black	438,478 (43.00%)	1,526,448 (21.96%)	1,154,286 (11.81%)	1,179,997 (8.80%)	
Others	95,496 (9.37%)	720,081 (10.36%)	987,170 (10.10%)	1,293,450 (9.65%)	
Education level					< 0.001
Less than high school	306,447 (30.05%)	1,588,058 (22.84%)	2,424,726 (24.81%)	2,138,734 (15.95%)	
High school or equivalent (including GED)	261,483 (25.65%)	1,733,434 (24.94%)	2,711,981 (27.76%)	3,117,715 (23.26%)	
College or above	451,720 (44.30%)	3,630,428 (52.22%)	4,634,430 (47.43%)	8,148,789 (60.79%)	
Smoking status					< 0.001
Never smoker	519,734 (50.97%)	3,679,383 (52.93%)	4,897,392 (50.12%)	6,585,262 (49.12%)	
Former smoker	238,436 (23.38%)	1,879,343 (27.03%)	3,340,785 (34.19%)	5,286,104 (39.43%)	
Current smoker	261,480 (25.64%)	1,393,194 (20.04%)	1,532,960 (15.69%)	1,533,872 (11.44%)	
BMI					< 0.001
≤ 24.9	71,964 (7.06%)	661,145 (9.51%)	1,120,509 (11.47%)	1,638,032 (12.22%)	
25–29.9	177,923 (17.45%)	1,462,864 (21.04%)	2,593,471 (26.54%)	4,281,873 (31.94%)	
> 30	769,763 (75.49%)	4,827,911 (69.45%)	6,057,157 (61.99%)	7,485,334 (55.84%)	
Hypertension					< 0.001
No	335,079 (32.86%)	2,179,028 (31.34%)	3,401,565 (34.81%)	3,231,156 (24.10%)	
Yes	684,571 (67.14%)	4,772,893 (68.66%)	6,369,572 (65.19%)	10,174,083 (75.90%)	
TC (mmol/L)	4.63 (3.61, 5.72)	4.81 (4.01, 5.61)	4.63 (3.96, 5.46)	4.53 (3.83, 5.33)	0.005
TG (mmol/L)	1.52 (1.07, 2.25)	1.81 (1.22, 2.70)	1.87 (1.24, 2.69)	1.78 (1.22, 2.61)	0.033
LDL (mmol/L)	2.45 (1.76, 3.22)	2.55 (1.93, 3.29)	2.52 (1.86, 3.17)	2.24 (1.69, 2.98)	< 0.001
HDL (mmol/L)	1.14 (0.96, 1.37)	1.14 (0.96, 1.36)	1.11 (0.96, 1.35)	1.22 (1.01, 1.50)	< 0.001
ALT (U/L)	19.00 (14.00, 30.00)	23.00 (17.00, 33.00)	23.00 (17.00, 32.00)	21.00 (16.00, 30.00)	0.007
AST (U/L)	20.00 (16.00, 28.00)	22.00 (18.00, 30.00)	22.00 (18.00, 28.00)	23.00 (18.00, 29.00)	0.043
UA (µmol/L)	339.00 (279.60, 410.40)	327.10 (273.60, 392.60)	327.10 (273.60, 398.50)	333.10 (273.60, 398.50)	0.5
FBG (mmol/L)	6.83 (5.83, 10.00)	7.60 (6.05, 11.10)	7.05 (5.89, 9.42)	6.66 (5.61, 8.55)	< 0.001
HBA1c (%)	7.00 (6.30, 9.00)	7.00 (6.30, 8.62)	6.70 (6.10, 7.80)	6.50 (5.90, 7.30)	< 0.001
BUN (mmol/L)	4.28 (3.21, 6.07)	4.64 (3.57, 5.71)	5.00 (3.93, 6.43)	5.71 (4.64, 7.50)	< 0.001
SCr (µmol/L)	72.49 (58.13, 91.94)	72.49 (60.11, 86.63)	75.14 (61.88, 90.39)	82.21 (68.07, 98.12)	< 0.001
UACR (mg/g)	17.04 (8.46, 90.47)	13.13 (6.76, 44.15)	11.22 (6.18, 28.06)	10.89 (6.12, 27.44)	< 0.001
DN					< 0.001
Non-DKD	636,764 (62.45%)	4,689,361 (67.45%)	7,494,128 (76.70%)	10,192,724 (76.04%)	
Microalbumin	239,482 (23.49%)	1,557,822 (22.41%)	1,775,327 (18.17%)	2,577,164 (19.23%)	
Macroalbumin	143,404 (14.06%)	704,737 (10.14%)	501,682 (5.13%)	635,351 (4.74%)	

Data are expressed as weighted number (weighted percentage) for categorical variables and median (interquartile range) for continuous variables

*n*, unweighted number; *N*, weighted number; *BMI*, body mass index; *TC*, total cholesterol; *TG*, triglyceride; *LDL*, LDL-cholesterol; *HDL*, HDL-cholesterol; *ALT*, alanine aminotransferase; *AST*, aspartate aminotransferase; *UA*, uric acid; *FBG*, fasting blood glucose; *HbA1c*, glycohemoglobin; *BUN*, blood urea nitrogen; *SCr*, serum creatinine; *UACR*, urine albumin to creatinine ratio

### Association between 25(OH)D3, 25(OH)D, and DN

Four logistic regression models were established to analyze the association between 25(OH)D3, 25(OH)D, and DN. Table 4 shows the association between 25(OH)D3, 25(OH)D, and DN in terms of odds ratio (OR) and 95% confidence interval (95% CI). Compared with the lowest quartile (Q1), 25(OH)D3 in the third and highest quartiles (Q3 vs Q1, OR = 0.61; 95% CI, 0.41–0.90; Q4 vs Q1, OR = 0.66; 95% CI, 0.46–0.95) were significantly associated with the decreased risk of DN in the unadjusted model (model 1). These results remained stable when we adjusted for age, sex, race, smoking status, education level, BMI, and hypertension (model 2, model 3). However, 25(OH)D3 only in Q4 was significantly associated with the decreased risk of DN in the fully adjusted model (model 4). In model 4, we found that the risk of DN was reduced by 31% with each unit increment of 25(OH)D3 level when 25(OH)D3 ≥ 75 nmol/L.

**Table 4:** Multivariable regression analyses of the association between serum level of 25(OH)D3, 25(OH)D, and the risk of DN

	Model 1	*P*-value	Model 2	*P*-value	Model 3	*P*-value	Model 4	*P*-value
	OR (95% CI)		OR (95% CI)		OR (95% CI)		OR (95% CI)	
25(OH)D3								
Q1	Reference		Reference		Reference		Reference	
Q2	1.02 (0.69,1.52)	> 0.9	1.05 (0.71,1.56)	0.8	1.09 (0.72,1.64)	0.7	1.13 (0.75,1.71)	0.5
Q3	0.61 (0.41,0.90)	0.014	0.60 (0.41,0.88)	0.009	0.63 (0.43,0.94)	0.025	0.71 (0.47,1.07)	0.1
Q4	0.66 (0.46,0.95)	0.025	0.59 (0.42,0.83)	0.003	0.62 (0.43,0.88)	0.01	0.69 (0.48, 0.99)	0.045
25(OH)D								
Q1	Reference		Reference		Reference		Reference	
Q2	0.80 (0.48,1.33)	0.4	0.78 (0.48,1.28)	0.3	0.79 (0.47,1.32)	0.4	0.79 (0.47,1.34)	0.4
Q3	0.51 (0.31,0.82)	0.006	0.46 (0.29,0.74)	0.002	0.48 (0.29,0.78)	0.004	0.51 (0.31,0.84)	0.009
Q4	0.52 (0.32,0.85)	0.009	0.44 (0.28,0.69)	< 0.001	0.45 (0.28,0.72)	0.001	0.48 (0.30,0.78)	0.004

Multivariable regression analyses were conducted to calculate weighted OR values

*Model 1*, adjusted for none; *model 2*, adjusted for age, sex, and race; *model 3*, model 2 + smoking status, education level, hypertension, and BMI; *model 4*, model 3 + TC, TG, HDL, ALT, UA, FBG, HbA1c, BUN, and SCr; *OR*, odds ratio; *CI*, confidence interval; *Q1*, quartile 1; *Q2*, quartile 2; *Q3*, quartile 3; *Q4*, quartile 4

Compared with the lowest quartile (Q1), 25(OH)D in the third and highest quartiles (Q3 vs Q1, OR = 0.51; 95% CI, 0.31–0.82; Q4 vs Q1, OR = 0.52; 95% CI, 0.32–0.85) were significantly associated with the decreased risk of DN in the unadjusted model (model 1). After adjusting for all covariates, 25(OH)D was consistently associated with the decreased risk of DN when 25(OH)D ≥ 50 nmol/L. Furthermore, in model 4, the risk of DN was reduced by 49% with each unit increment of 25(OH)D level when 50 nmol/L ≤ 25(OH)D < 75 nmol/L. When 25(OH)D ≥ 75 nmol/L, the risk of DN was reduced by 52% with each unit increment of 25(OH)D level. Therefore, it can be concluded that high doses of VD have a protective effect on DN.

### The effects of Tan on the general conditions of DN rats

The general conditions of the rats in each group were observed daily during administration. Compared with the control group, the rats in the DN group showed an obvious increase in water intake (about 4–5 times that of the control group), food intake, and urine output (the bedding was wet or even oozing, requiring daily replacement). Also, the rats were skinny, greasy, and in poor spirits. The general conditions of rats in DN + Tan1, DN + Tan2, and DN + Par groups were basically the same as those in the DN group from 0 to 28 days. From the 28th day of administration, the “three more and one less” symptoms in rats in DN + Tan1, DN + Tan2, and DN + Par groups were slightly improved. At the end of the experiment, all 50 rats survived, and one rat in the DN + Par group showed a cataract.

**Fig. 3:**
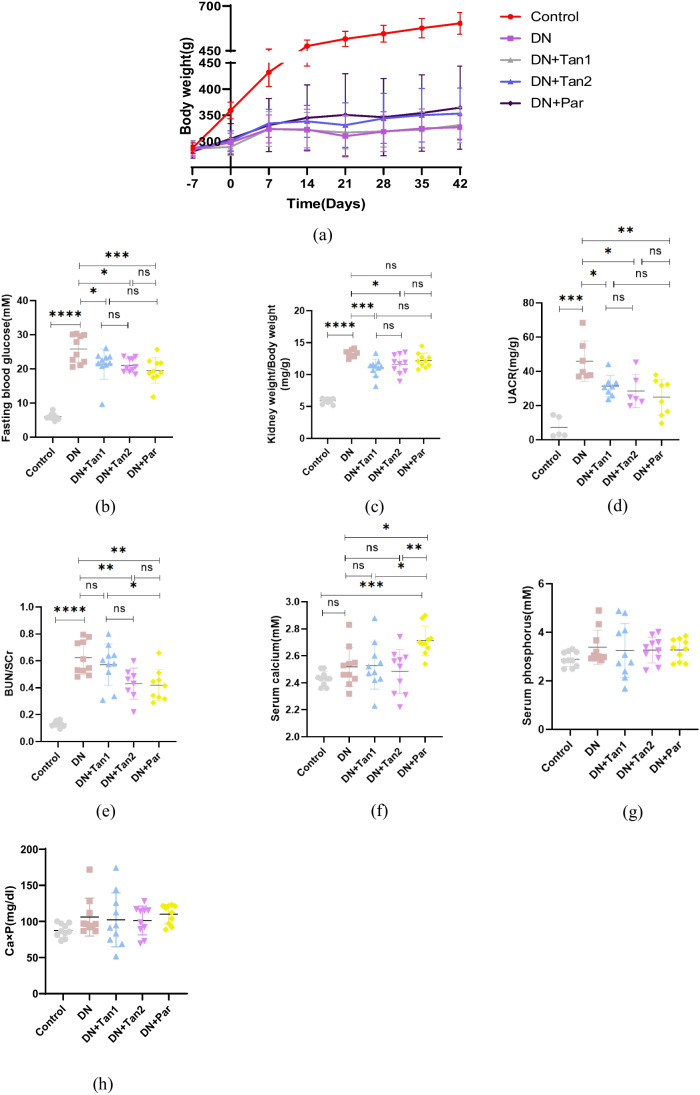
The effects of Tan (5.4 mg/kg), Tan (10.8 mg/kg), and Par (0.054 µg/kg) on body weight, fasting blood glucose, renal function, and calcium and phosphorus metabolism in DN rats. **a** Body weight. **b** Fasting blood glucose. **c** Renal index (kidney weight/body weight). **d** Urine albumin to creatinine ratio (UACR). **e** BUN/Scr. **f** Serum calcium. **g** Serum phosphorus. **h** Calcium-phosphorus product. Control, control group; DN, diabetic nephropathy group; DN + Tan1, diabetic nephropathy with administration of Tan at 5.4 mg/kg group; DN + Tan2, diabetic nephropathy with administration of Tan at 10.8 mg/kg group; DN + Par, diabetic nephropathy with administration of Par at 0.054 µg/kg group. *n* = 10 rats per group. ^*^P < 0.05, ^**^P < 0.01, ^***^P < 0.001, ^****^P < 0.0001. *n* = 10 per group (the UACR index could not be detected in some rats because urine was not obtained by bladder puncture. UACR, *n* = 5, 7, 8, 6, 8, respectively)

As shown in Fig. 3a, the rats in the control group showed a steady increase in body weight each week. However, the rats in the DN group exhibited significant wasting compared with the control group. There were no significant changes in the body weight of rats after the injection of Tan (5.4 mg/kg), while the rats showed a gradual increase in body weight after the injection of Tan (10.8 mg/kg) and Par (0.054 µg/kg), but the results were not statistically different.

The levels of FBG of rats in the DN group were significantly higher than those in the control group (*P* < 0.0001). After 42 days of administration, the FBG levels of rats in the DN + Tan1 group (*P* < 0.05), DN + Tan2 group (*P* < 0.05), and DN + Par (*P* < 0.001) group were significantly decreased compared with the DN group (Fig. 3b).

### The effects of Tan on the renal function in DN rats

As shown in Fig. 3c, the renal index (kidney weight/body weight) of rats in the DN group was significantly higher than that in the control group (*P* < 0.0001). Compared with the DN group, the renal indexes in the DN + Tan1 group and DN + Tan2 group were significantly improved (*P* = 0.0009 and *P* = 0.0176, respectively), while there were no significant differences in renal indexes between the DN and DN + Par group (*P* = 0.2622).

Compared with the control group, the blood samples of rats in the DN group showed significant renal function impairment with increased levels of BUN/SCr. There were no significant differences in BUN/SCr levels between the DN group and the DN + Tan1 group. The levels of BUN/SCr of rats in DN + Tan2 and DN + Par groups were significantly decreased compared with those in the DN group (Fig. 3e).

Furthermore, the UACR level in the DN group was significantly higher than that in the control group (*P* < 0.001). After 42 days of administration, the UACR level in the DN + Tan1 group (*P* < 0.05), DN + Tan2 group (*P* < 0.05), and DN + Par (*P* < 0.01) group was significantly reduced compared with the DN group, and the reduction of UACR in the DN + Par group was the most significant (Fig. 3d).

### The effects of Tan on the calcium and phosphorus metabolism in DN rats

The serum calcium level of rats receiving Par was significantly increased. Except for that, there were no statistical differences in the levels of serum calcium, phosphorus, and calcium-phosphorus product among these five groups (Fig. 3 f–h).

### The effects of Tan on the renal pathological changes in DN rats

**Fig. 4:**
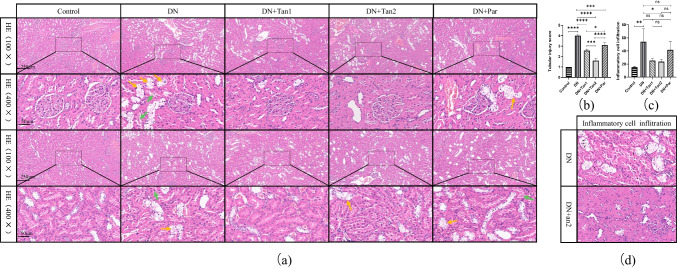
The effects of Tan (5.4 mg/kg), Tan (10.8 mg/kg), and Par (0.054 µg/kg) on the HE staining in DN rats. **a** Pathological changes of glomeruli and tubules in HE staining sections at 100 × and 400 × . **b** Tubular injury score, analysis of HE staining results of renal tissue in five groups. **c** Quantitative analysis of inflammation cell infiltration. **d** HE staining of inflammatory cell infiltration. Yellow arrows, tubular epithelial cells with vacuolar and particle denaturation. Green arrows, cell fragments or tube-type cells. Black arrows, inflammation cell infiltration

To reveal the effects of Tan on the renal pathological changes in DN rats, HE staining was performed and observed under the microscope. As shown in Fig. 4a, normal morphology and size of glomeruli in the control group were observed, with no mesangial cells or stromal proliferating, no cystic wall thickening, and no crescent formation. Meanwhile, the tubular lumen of the control group had no atrophy or dilatation, no vacuolar degeneration, no cell fragments or tube-type cells in the lumen, brush border visible in the proximal tubule, and normal morphology in the distal tubule and collecting duct. Compared with the control group, the DN group showed irregular glomerular morphology, marked proliferation of mesangial cells, disordered arrangement of renal tubules, obvious swelling of tubular epithelial cells with vacuolar and particle denaturation (Fig. 4a, yellow arrows), detachment of tubular brush border, formation of cell fragments or even tube-type cells (Fig. 4a, green arrows), infiltration of focal lymphatic, and neutrophil in the renal interstitium (Fig. 4d, black arrows). After the treatment of Tan and Par, the above pathological changes in the kidneys of rats were alleviated compared with those in the DN group. Among them, the renal pathological damage in the rats receiving Tan (10.8 mg/kg) was most significantly alleviated, but the three groups failed to recover to the normal physiological state of the control group. Vacuolar degeneration, cell fragments, and tube-type cells were still present in all three treatment groups after administration, but the degree of damage was significantly reduced compared to the DN group. The rats treated with 10.8 mg/kg Tan had the least vacuolar degeneration, the least proportion of tubular dilatation, significantly reduced cell fragments and tube-type cells, and significantly improved inflammatory cell infiltration.

**Fig. 5:**
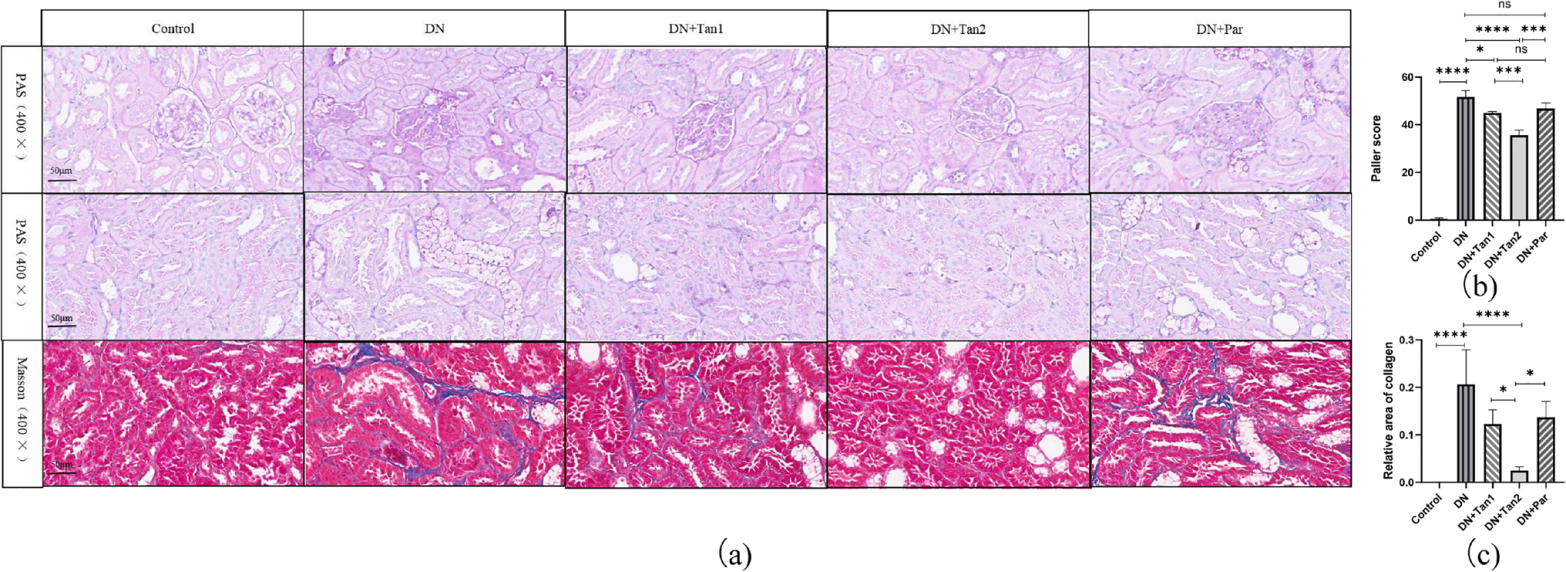
The effects of Tan (5.4 mg/kg), Tan (10.8 mg/kg), and Par (0.054 µg/kg) on the renal pathological changes in DN rats. The STZ induced severe renal damage, which could be alleviated by Tan (10.8 mg/kg). **a** The effects of Tan (5.4 mg/kg), Tan (10.8 mg/kg), and Par (0.054 µg/kg) on the PAS and Masson staining in DN rats. **b** Paller score, analysis of PAS staining results of renal tissue in five groups. **c** Relative area of collagen, the ratio of the blue area to the visual field area in Masson staining. ^*^P < 0.05, ^***^P < 0.001, ^****^P < 0.0001

To further evaluate the effects of Tan on tubular injury and tubulointerstitial fibrosis, PAS and Masson staining were performed on kidney specimens. As shown in Fig. 5a, the results of PAS staining were consistent with those of HE staining. Typical pathological changes were observed in the glomerulus and renal tubules in the DN group, including morphological changes of the glomerulus, thickening of the glomerular basement membrane, the proliferation of mesangial cells, a large amount of glycogen deposition, vacuolation of renal tubules, and detachment of brush border of the renal tubule. After Tan (low and high dose) intervention, the pathological changes of glomeruli and renal tubules were alleviated, while the glomeruli and renal tubules were not significantly improved in the DN + Par group. In addition, Masson staining displayed that, compared with the control group, obvious blue collagen deposition was seen in the DN group, indicating that the DN model was successfully established. After 42 days of administration of Tan (5.4 mg/kg, 10.8 mg/kg) and Par (0.054 µg/kg), the degree of tubulointerstitial fibrosis in rats was improved. According to the deposition level of collagen, high-dose Tan had the most obvious protective effect on renal fibrosis in DN rats, followed by low-dose Tan, and Par had the weakest protective effect (Fig. 5c).

To intuitively show that Tan alleviates renal pathological damage in DN rats, a semi-quantitative analysis was performed on the results of HE staining. As shown in Fig. 4b, compared with the control group, renal tubules in the DN group were severely damaged (*P* < 0.0001). Tan (5.4 mg/kg and 10.8 mg/kg) and Par (0.054 µg/kg) significantly improved tubular injury in DN rats (*P* < 0.0001, *P* < 0.0001, *P* < 0.001, respectively). To better compare the efficacy of drugs and the pathological changes in rats after administration, we further supplemented the Paller score for PAS staining. As presented in Fig. 5b, the Paller score was higher in the DN group at 51.63 ± 2.72 versus 0.60 ± 0.35 in the control group (*P* < 0.0001). After 42 days of administration of Tan (5.4 mg/kg, 10.8 mg/kg) and Par (0.054 µg/kg), the Paller score decreased significantly, indicating the improvement of pathological changes. Among them, high-dose Tan had the most significant improvement with a Paller score of 35.70 ± 2.14, low-dose Tan was second with a Paller score of 45.03 ± 0.50, and Par had the weakest improvement with a Paller score of 46.83 ± 2.40.

In addition to the above two semi-quantitative analyses of tubular injury performed on HE staining and PAS staining, respectively, we also performed a quantitative analysis of HE staining. We counted inflammatory cells including plasma cells, lymphocytes, monocytes, neutrophils, and eosinophils. The results showed significant inflammatory cell infiltration in the DN group, while inflammatory cell infiltration was significantly improved after high concentration of Tan administration (Fig. 4c). These data supplied strong evidence to support the conclusion that Tan may attenuate the renal fibrosis caused by 1% STZ.

### Tan ameliorates tubulointerstitial fibrosis through regulation of VDR/Wnt/β-catenin pathway

**Fig. 6:**
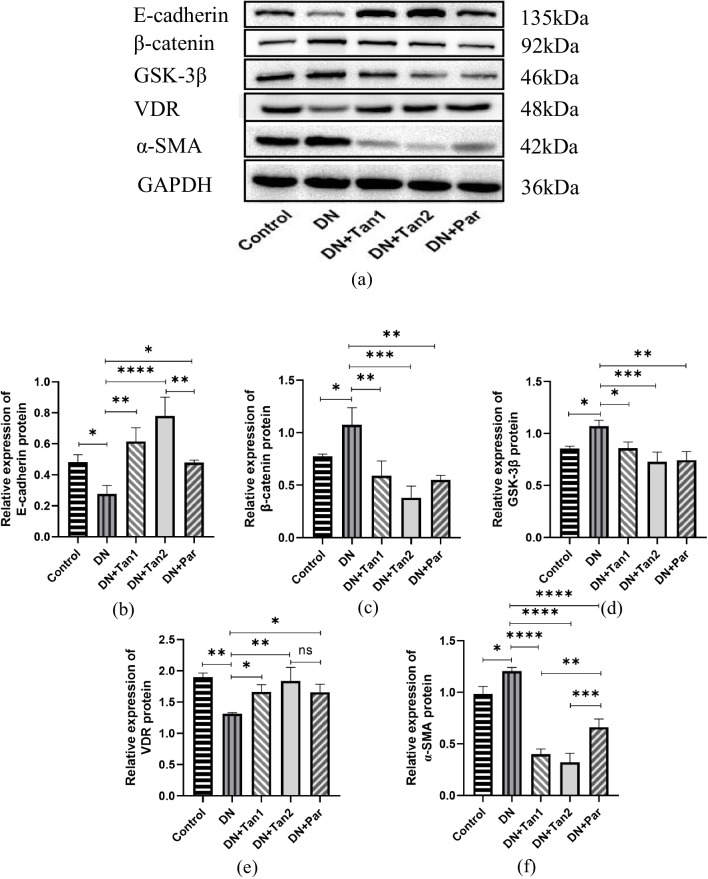
The effects of Tan (5.4 mg/kg), Tan (10.8 mg/kg), and Par (0.054 µg/kg) on the renal fibrosis-related protein expression in DN rats. Tan (10.8 mg/kg) was considered to alleviate renal fibrosis by regulating VDR/Wnt/β-catenin. **a** Renal fibrosis-related protein expression in five groups. Compared with the control group, α-SMA, β-catenin and GSK-3β levels in the DN group were significantly increased, while E-cadherin and VDR levels were significantly decreased. Compared with the DN group, the α-SMA, β-catenin, and GSK-3β levels were significantly lower, while E-cadherin and VDR levels were significantly higher in the three groups. The relative protein expression levels of **b** E-cadherin, **c** β-catenin, **d** GSK-3β, **e** VDR, and **f** α-SMA were evaluated by GraphPad. ^*^P < 0.05, ^**^P < 0.01, ^***^P < 0.001, ^****^P < 0.0001. The experiments were repeated three times using different samples

As shown in Fig. 6a, b, and f, compared with the control group, E-cad protein expression levels in the DN group were significantly decreased (0.48 ± 0.05 vs 0.28 ± 0.05, *P* < 0.05), while α-SMA protein expression levels were significantly increased (0.98 ± 0.07 vs 1.20 ± 0.04, *P* < 0.05). It was suggested that STZ induced renal fibrosis in DN rats. After the intervention, the renal fibrosis levels of rats in three groups were improved to varying degrees. Compared with the DN group, the expression levels of E-cad protein were significantly increased after drug treatment (0.28 ± 0.05 in DN vs 0.62 ± 0.09 in DN + Tan1; 0.78 ± 0.12 in DN + Tan2; 0.48 ± 0.02 in DN + Par), while α-SMA protein expression levels were significantly decreased (1.20 ± 0.04 in DN vs 0.40 ± 0.05 in DN + Tan1; 0.32 ± 0.09 in DN + Tan2; 0.66 ± 0.08 in DN + Par). The results showed that among the three drugs, high-dose Tan was the most effective in alleviating renal fibrosis in DN rats, low-dose Tan was inferior to high-dose Tan, and Par was the weakest. This indicated that both Tan and Par had protective effects on DN, and the protective effect of Tan was dose-dependent.

To further investigate the mechanism of renal fibrosis in DN rats, the protein expression levels of β-catenin, GSK-3β, and VDR were detected by western blotting. As shown in Fig. 6a and c–e, compared with the control group, the protein levels of β-catenin (0.77 ± 0.02 vs 1.07 ± 0.16) and GSK-3β (0.85 ± 0.03 vs 1.07 ± 0.06) in the DN group were significantly increased, while the protein levels of VDR (1.90 ± 0.07 vs 1.31 ± 0.02) were significantly decreased. Compared with the DN group, the protein levels of β-catenin (1.07 ± 0.16 in DN vs 0.59 ± 0.14 in DN + Tan1; 0.38 ± 0.11 in DN + Tan2; 0.55 ± 0.04 in DN + Par) and GSK-3β (1.07 ± 0.16 in DN vs 0.86 ± 0.06 in DN + Tan1; 0.73 ± 0.09 in DN + Tan2; 0.74 ± 0.08 in DN + Par) were significantly lower and VDR protein expression levels (1.31 ± 0.02 in DN vs 1.67 ± 0.10 in DN + Tan1; 1.84 ± 0.22 in DN + Tan2; 1.66 ± 0.13 in DN + Par) were significantly higher in the three groups, suggesting that the VDR/Wnt/β-catenin signaling pathway was involved in the occurrence and development of tubulointerstitial fibrosis in rats with diabetic nephropathy.

## Discussion

Diabetes is a metabolic disease involving multiple systems in the whole body. As one of the serious microvascular complications of diabetes, DN has a complex pathogenesis, and the various factors and signaling pathways involved have not yet been identified. Excessive glucose and lipid levels caused by disorders of glucose and lipid metabolism, renal hemodynamic alterations, autophagy, oxidative stress, endoplasmic reticulum stress, inflammatory mechanisms, and genetics are all considered to promote the progression of DN (Tuttle et al. 2022b; DeFronzo et al. 2021; Dagar et al. 2021). In previous studies on the pathogenesis of DN, the part that has attracted the most attention is the activation of inflammatory response. It was found that the inflammatory response could be significantly alleviated by hydrolyzing S-adenosylhomocysteine (Dai et al. 2021), administering hypoxia-inducible factor (HIF) stabilizers (Hasegawa et al. 2020), inhibiting inflammatory factors (Melo et al. 2022), increasing the expression of annexin A1 (Wu et al. 2021), and up-regulating the expression of miR-499-5p (Zhou et al. 2021). The oxidative stress in the pathogenesis of DN is also of equal concern with the inflammatory response (Zhong et al. 2022). A variety of drugs have been tried to inhibit the expression of superoxide dismutase (SOD) and malondialdehyde (MDA) to suppress oxidative stress. Among them, diphenyl diselenide (Wang et al. 2021), crocin (Ali Hammood Keelo et al. 2022), and other drugs have been proved by animal experiments to reduce oxidative stress and improve renal dysfunction. In addition, autophagy, disorder of glucose and lipid metabolism, and genetics mentioned above also play an important role in the pathogenesis of DN, and these mechanisms interact with each other constantly.

The severity of renal impairment in DN patients is assessed by symptoms, renal function indicators, and pathological changes. In terms of clinical manifestations, DN patients have lower renal function such as elevated serum creatinine, urea, urinary albumin, and UACR, in addition to the main manifestations of diabetes mellitus such as hyperglycemia, polyphagia, polydipsia, polyuria, and wasting. In terms of pathological changes, in the past, DN was considered a disease characterized mainly by glomerular injury, while the role of tubular injury in the occurrence and development of the disease has been neglected for a long time. Recently, more and more studies have revealed the importance of tubulointerstitial injury in the progression of DN (Collins et al. 2022; Li et al. 2019, 2020; Chen et al. 2020). Teumer et al. (Teumer et al. 2016) conducted a genome-wide association analysis on a large sample (2,191,945 single nucleotide polymorphic loci in 54,450 participants) and demonstrated that mutation of Rab38 gene (expressed in proximal tubular cells) in DN patients may increase the UACR level, which is mainly due to the impaired function of the protein encoded by Rab38 gene regulating vesicular transport, affecting the excretion of patients’ urinary microalbumin. Artur et al. (Rangel-Filho et al. 2013) studied hypertension-associated nephropathy rats with Rab38 knockout and found that the reason why the rats have elevated UACR level and impaired renal function may be that the dysfunction of renal tubular reabsorption caused by Rab38 knockout, while glomerular injury has little effect in this process. We therefore speculate that Rab38 gene cannot be normally expressed in the renal tubules of patients with DN, resulting in a significant decline in renal function, while the reduction of Rab38 expression further aggravates renal tubule damage, thus increasing the UACR level.

In this study, we successfully established the DN model by injecting STZ. STZ-induced DN rats showed obvious symptoms of diabetes; meanwhile, there is a significant decline in renal function in DN rats, including significantly increased levels of kidney weight/body weight, BUN/SCr, and UACR. In addition, the renal pathology of DN rats showed obviously damaged glomeruli and tubules, with typical TIF pathological changes. The destruction of glomerular basement membrane (GBM), the damage of filtration function of GBM, and the reabsorption disorder of renal tubules lead to the accumulation of urea in the body and the increase of UACR. The levels of serum urea and creatinine are affected by many factors such as food intake and body weight, and it is difficult to accurately reflect the level of renal function. As an early detection indicator of DN, the increase of UACR level is of great significance for the early clinical detection, early treatment of DN, and early secondary prevention. Although there is a significant positive correlation between UACR levels and degree of DN renal pathology, it is not strictly proportional, and it is not uncommon for UACR to be normal in patients with diabetic nephropathy (Sasaki et al. 2021). Therefore, we should combine the renal function level and renal pathology to make a comprehensive judgement of the condition.

The decreased expression level of E-cad protein and the increased expression level of α-SMA protein in renal tissue indicate the occurrence of EMT and the intensification of renal fibrosis. Interestingly, studies have found that renal fibrosis occurs when almost all chronic kidney diseases, including DN, progress to the end-stage, and it is the pathological basis of ESRD. Activation of the above-mentioned pathogenesis of DN can promote renal fibrosis and lead to renal dysfunction. Many studies have tried to elucidate the mechanism of renal fibrosis, but there is no definite conclusion at present. Renal fibrosis is mainly manifested by TIF and glomerulosclerosis, during which a large amount of extracellular matrix (ECM) is deposited and EMT occurs (Gui et al. 2021). Our results showed that renal tissue derived from DN rats had significant EMT and TIF. We found that the more serious the TIF is, the more severe the DN lesions are. In other words, improving TIF to delay the progression of renal fibrosis may significantly improve DN and even treat other chronic kidney diseases. Therefore, in this study, we tried to find a drug that can improve TIF and alleviate renal fibrosis to relieve DN.

Song et al. (Song et al. 2020) found that Tan could resist colorectal cancer metastasis by inhibiting the expression of EMT-related molecules in human colorectal cancer cell lines. Fu et al. 2021 investigated that Tan protected purulent endometritis by suppressing lipopolysaccharide-induced EMT in bovine endometrial epithelial cells. Therefore, we hypothesized that Tan could reduce DN-induced renal dysfunction and TIF by inhibiting the expression of EMT-related molecules. Although Tan is currently mainly used in the clinical treatment of cardiovascular diseases and has not been used in the clinical treatment of DN, it has been reported to have the effects of improving blood glucose, anti-inflammation, and anti-fibrosis. VDR agonist Par, a vitamin D analogue, was found to attenuate TGF- β1-induced EMT of human peritoneal mesothelial cells and protect the peritoneum from EMT and fibrosis (Ko et al. 2019). Lim et al. 2021 found that Par could improve HIF-1α-induced and TGF-β1-induced damage of kidney pericytes in mice, thereby improving renal fibrosis and delaying the progression of DN to ESRD. Therefore, Par was used as a positive control drug in this study to observe the changes of TIF-related indexes in DN rats after treatment with low and high doses of Tan and Par.

Our study on NHANES has shown that participants with higher 25(OH)D levels were more likely to have lower levels of TC, TG, LDL, HDL, ALT, AST, FBG, HbA1c, and UACR. The results of our animal experiments suggested that both Tan and Par could protect DN rats by improving FBG, UACR, and BUN/SCr, reducing renal fibrosis, and inhibiting EMT by regulating VDR/Wnt/β-catenin pathway. By comparing the renal protective effects of these drugs, it is obviously found that high-dose Tan is stronger than low-dose Tan, and Par is the weakest among them. At present, there is no drug that can reverse fibrosis, so it is important to intervene in the early stage of DN to prevent the formation of fibrosis in time. Due to the progression of renal fibrosis in DN rats, obvious EMT occurs in renal tissue, accompanied by the constant replacement of the tubular epithelial cells by mesenchymal cells. The dysfunction of renal tubules led to the increase of UACR and TIF. On the contrary, Tan and Par administration at the early stage of DN in rats could significantly improve EMT, reduce the formation of mesenchymal cells, and prevent further deterioration of renal function.

In this study, after adjustment for all confounders, we found that the prevalence and severity of DN in diabetic patients were strongly associated with 25(OH)D and 25(OH)D3. When 50 nmol/L ≤ 25(OH)D < 75 nmol/L, 25(OH)D, the most reliable indicator of VD, decreased the risk of DN by 49% for each unit increase in its level, and when 25(OH)D ≥ 75 nmol/L, it decreased the risk of DN by 49% for each unit increase in its level. In addition, Tan and Par alleviated DN rats’ kidney damage by activating VDR, which revealed that the lack of VDR is crucial in the occurrence of TIF in DN. It is basically consistent with the experimental results of Li et al. (Li et al. 2022). Li established DN models in VDR knockout mice and VDR overexpression mice, respectively, and then used Par to treat in VDR knockout DN mice. It was found that both Par intervention and VDR overexpression could reduce urinary albumin excretion and improve renal tubule damage and inflammatory response in DN mice. However, Li’s study found that Par could not significantly reduce blood glucose, so whether Par could improve blood glucose level remains controversial. An observational study showed that VD deficiency and VDR deficiency were associated with diabetes and other metabolic diseases (Rosen et al. 2012). It has been confirmed that VDR loss occurs in the early stage of unilateral ureteral obstruction model in mice and early renal biopsy in patients (including patients with minimal change nephropathy, DN, and hypertensive nephropathy). Moreover, the administration of vitamin D to mice on the fifth day after the establishment of ureteral obstruction model could restore the expression of VDR and inhibit inflammatory reaction, thereby significantly reducing EMT and improving renal fibrosis (Xiong et al. 2012). It is reported that Par reduced renal inflammation in mice with obstructive nephropathy and inhibited the expression of RANTES only expressed in renal tubules, thus achieving the anti-fibrosis effect (Tan et al. 2008).

A randomized double-blind crossover trial suggested that Par may cause calcium and phosphorus metabolism disorder after long-term medication (Parvanova et al. 2018), but this problem exists commonly in the clinical application of VDR agonists. Therefore, we expect to find drugs that can not only increase the level of VDR, but also have better efficacy and fewer side effects on DN. In this experiment, Tan was applied to treat DN rats, and the protective effects of Tan and Par were compared. The experimental results revealed that Tan was significantly stronger than Par in reducing renal weight/body weight and improving renal fibrosis in DN rats. Meanwhile, Tan has a more significant role in alleviating EMT by regulating the VDR/Wnt/β-catenin pathway. We speculate that there may be two reasons why the efficacy of Par is inferior to Tan. First, Par can only exclusively activate VDR, while Tan may protect DN by activating other mechanisms besides VDR. Second, we believe that the dosage of drugs has also played an important role.

Wnt/β-catenin signaling pathway plays an important role in vascular diseases such as myocardial infarction (He et al. 2022), atherosclerosis (Zang et al. 2022), cardiovascular calcification (Wang et al. 2019), and diabetic retinopathy [44[. Besides, the activation of Wnt/β-catenin pathway when EMT and TIF occur has been proved in a lupus nephritis model (Fu et al. 2019). Notably, our previous experiments confirmed that elevated VDR improved EMT of HK-2 cells by downregulating Wnt/β-catenin pathway (Zeng and Bao 2021). Muralidhar et al. (Muralidhar et al. 2019) studied murine melanoma cells and found that Wnt/β-catenin signaling pathway was significantly inhibited when VDR was highly expressed, which is consistent with our research. Consequently, VDR/Wnt/β-catenin pathway has great potential to become an effective target for the treatment of DN.

There are still some limitations in this study. Firstly, the NHANES study was cross-sectional, and we still need cohort studies to further clarify the causal relationship between VD and DN. Secondly, Tan and Par have a short intervention time in the animal experiment, and their long-term effects on DN rats have not been observed. In future experiment, the drug intervention time can be extended to further evaluate the protective effect and adverse effects of drugs on DN. Thirdly, only α-SMA and E-cad were used to evaluate the degree of fibrosis, which could be evaluated by more other indicators such as fibronectin and collagen IV. Fourthly, we speculate that Tan can improve DN fibrosis not only by regulating VDR/Wnt/β-catenin pathway, but also by regulating other pathways, which still needs further study. Finally, it has been confirmed in animal experiments that Tan has a certain protective effect on DN rats, and the clinical efficacy of Tan on DN patients can be further explored in the future.

Taken together, Tan is superior to paricalcitol in ameliorating tubulointerstitial fibrosis in DN rats, which can improve the symptoms of “three more and one less,” reduce FBG level, improve renal index, BUN/Scr, and UACR, reduce histopathological damage of kidney, and improve the expression of fibrosis-related proteins in kidney tissue by regulating VDR/Wnt/β-catenin pathway.

### Supplementary Information

Below is the link to the electronic supplementary material.

Supplementary file1 (DOC 3933 KB)

## Data Availability

The datasets used and/or analyzed during the current study are available from the corresponding author on reasonable request.

## Abbreviations

VDR: Vitamin D receptor
TIF: Tubulointerstitial fibrosis
DN: Diabetic nephropathy
EMT: Epithelial-to-mesenchymal transition
Tan: Tanshinone IIA
ESRD: End-stage renal disease
Par: Paricalcitol
FBG: Fasting blood glucose
SCr: Serum creatinine
UACR: Urine albumin to creatinine ratio
H&E: Hematoxylin and eosin
PAS: Periodic acid-Schiff
SOD: Superoxide dismutase
MDA: Malondialdehyde
GBM: Glomerular basement membrane
ECM: Extracellular matrix
