# Supplementary material for: Tanshinone IIA is superior to paricalcitol in ameliorating tubulointerstitial fibrosis through regulation of VDR/Wnt/β-catenin pathway in rats with diabetic nephropathy
Source: Naunyn Schmiedebergs Arch Pharmacol. 2023 Nov 22;397(6):3959–77. doi: 10.1007/s00210-023-02853-3 (PMC11111530; doi:10.1007/s00210-023-02853-3)
Supplement: Supplementary file 1 — Supplementary file1 (DOC 3933 KB) [file 210_2023_2853_MOESM1_ESM.doc]

# Tanshinone IIA is superior to paricalcitol in ameliorating tubulointerstitial fibrosis through regulation of VDR/Wnt/β-catenin pathway in rats with diabetic nephropathy

Jing-Yi Zeng^a,b^, Yu Wang^b^, Fu-Yuan Hong^a^, Miao Miao^b^, Yu-Ying Jiang^b^, Zi-Xuan Qiao^b^, Yun-Tao Wang^b^, Xiao-Rong Bao^b*^

^a^Department of Nephrology, Fujian Provincial Hospital, Shengli Clinical Medical College of Fujian Medical University, Fuzhou, China.

^b^Department of Nephrology, Jinshan Hospital of Fudan University, Shanghai, China.

Short Title: Tanshinone IIA ameliorates tubulointerstitial fibrosis

*** Corresponding Author:**

Xiao-Rong Bao

Department of Nephrology

Jinshan Hospital of Fudan University

Shanghai, China.

Tel: 18930819120

1. mail: [xrbao19660108@163.com](mailto:xrbao19660108@163.com)


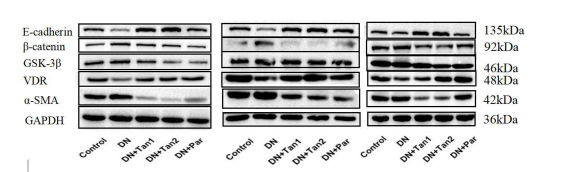


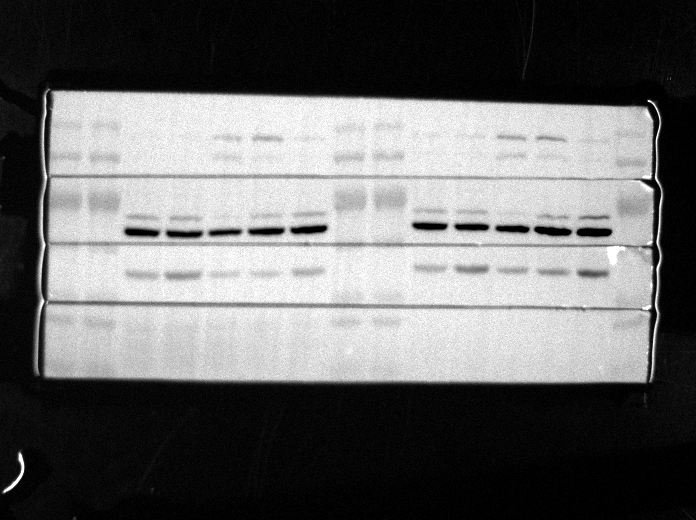


E-cadherin

α-SMA

GAPDH

MMP-9

92kDa

84kDa

135kDa

42kDa

36kDa


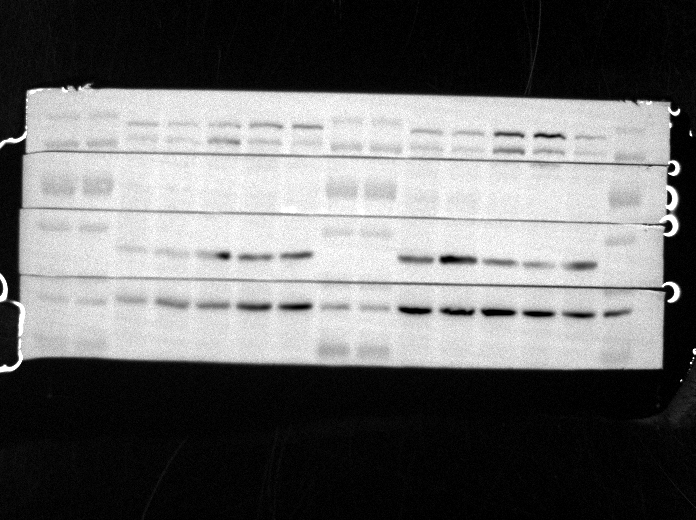


E-cadherin

α-SMA

GAPDH

β-catenin

92kDa

135kDa

42kDa

36kDa

E-cadherin 135kDa


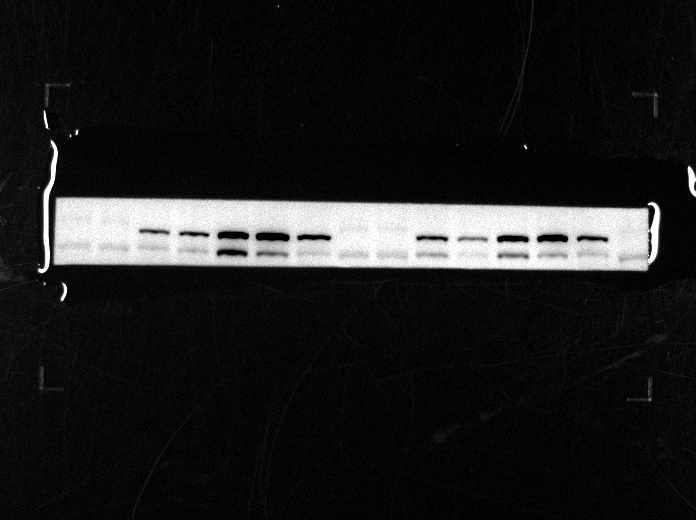


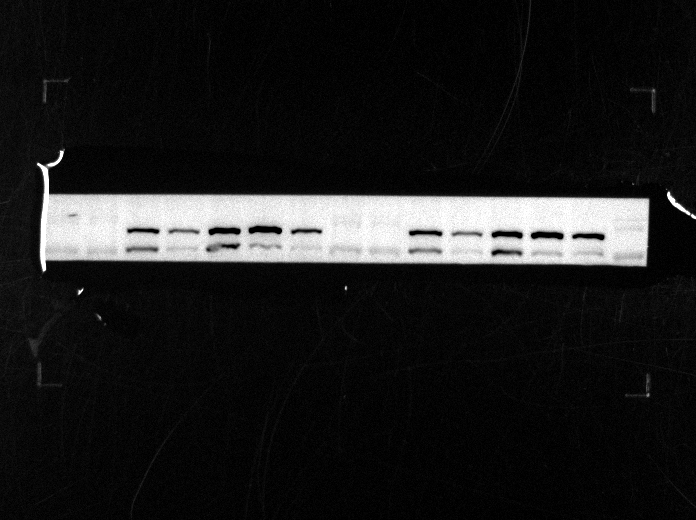


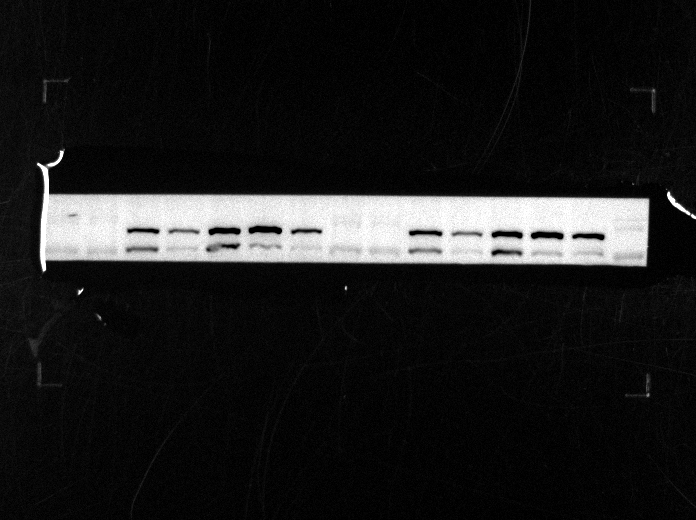


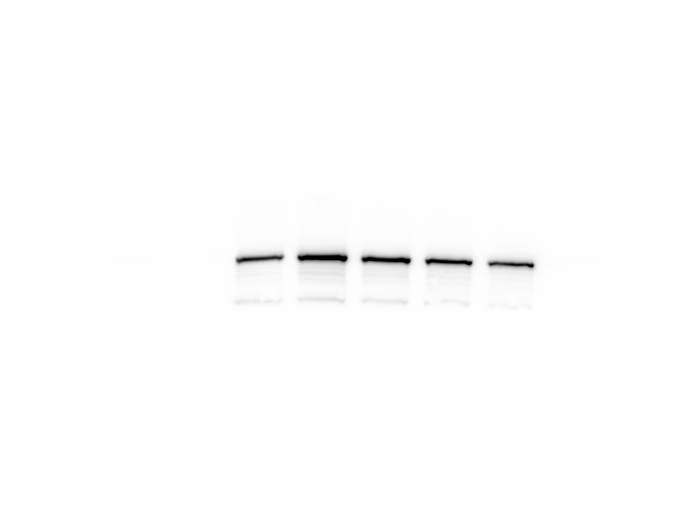
β-catenin 92kDa


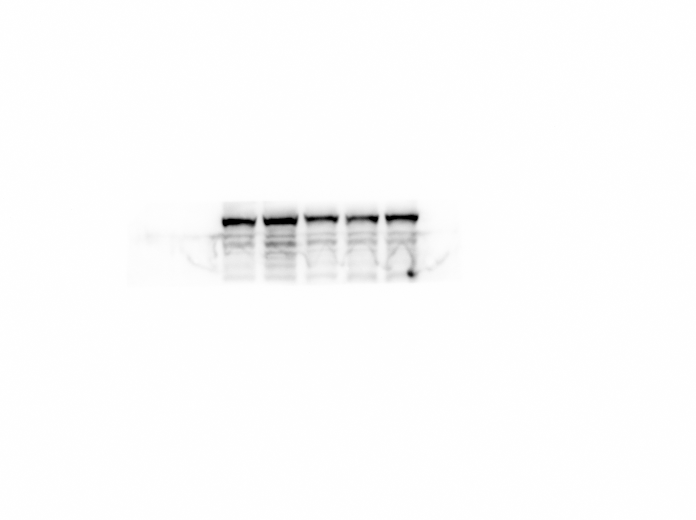


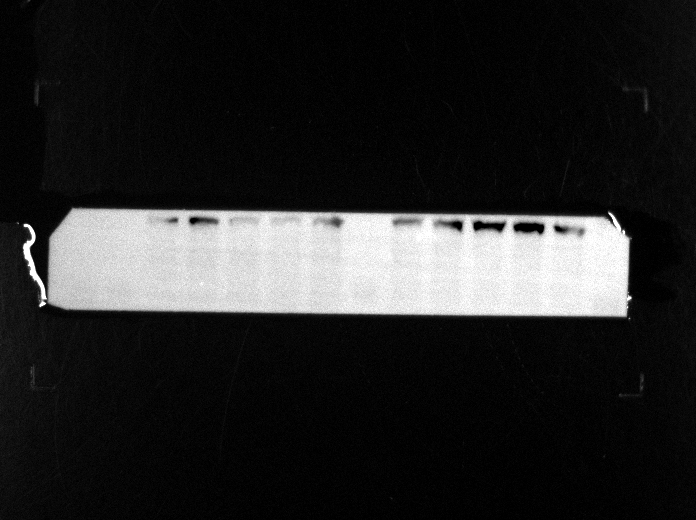


GSK-3β 46kDa


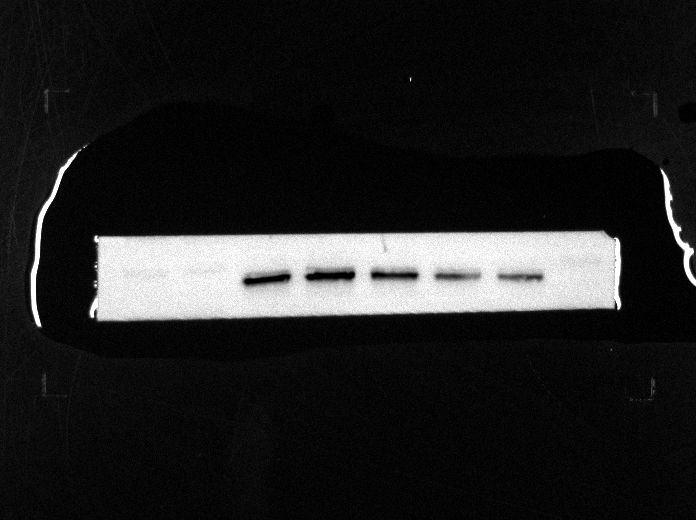


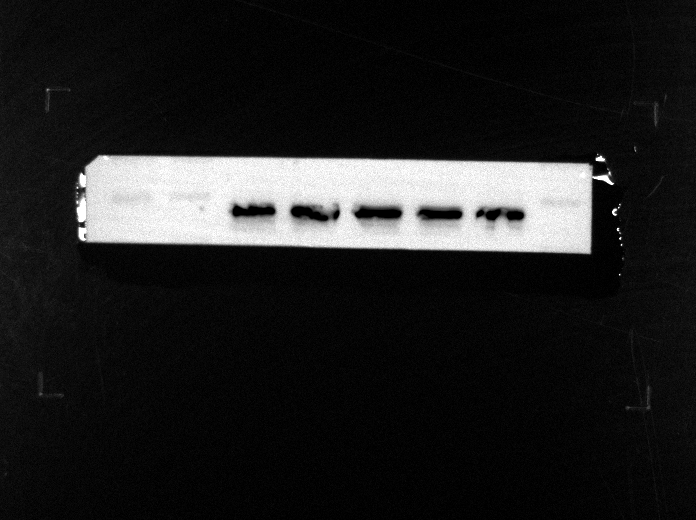


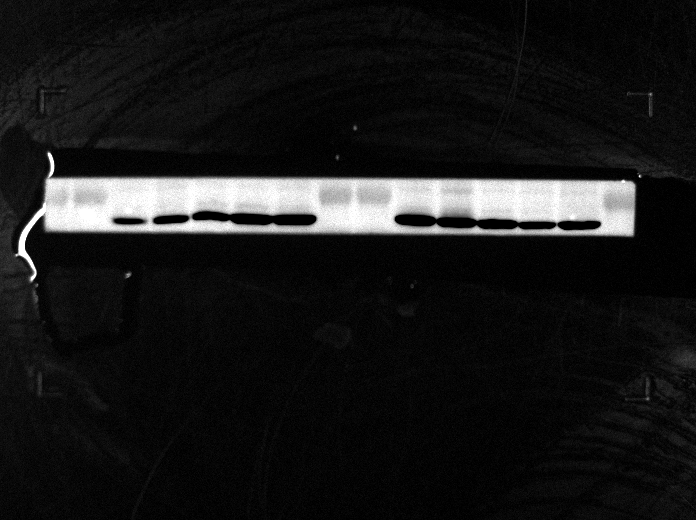


VDR 54kDa

48kDa


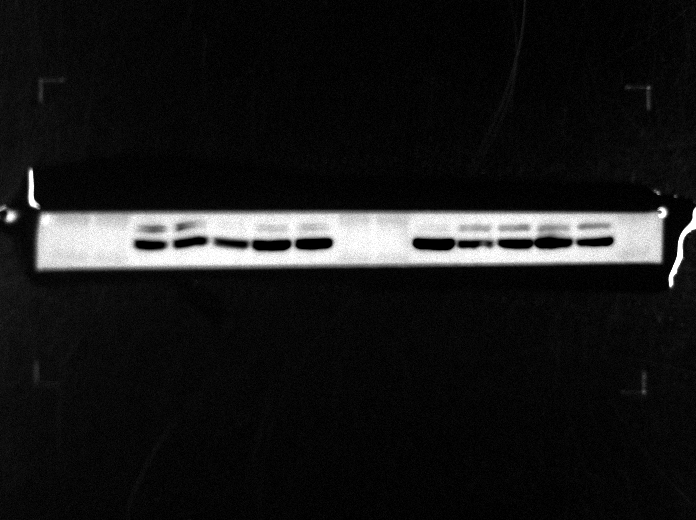


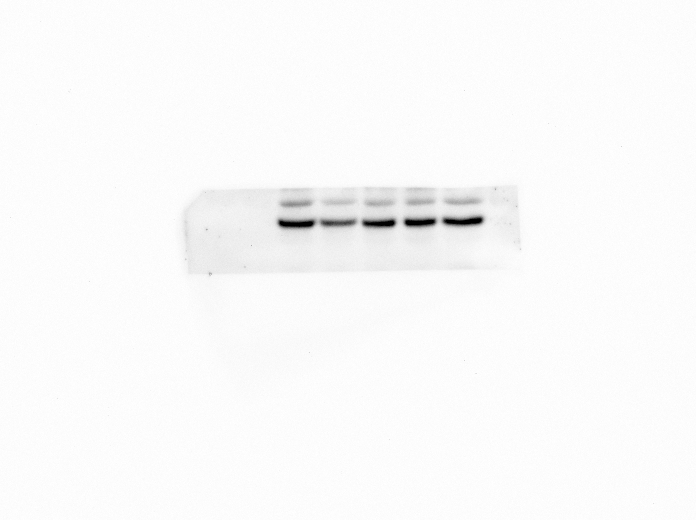


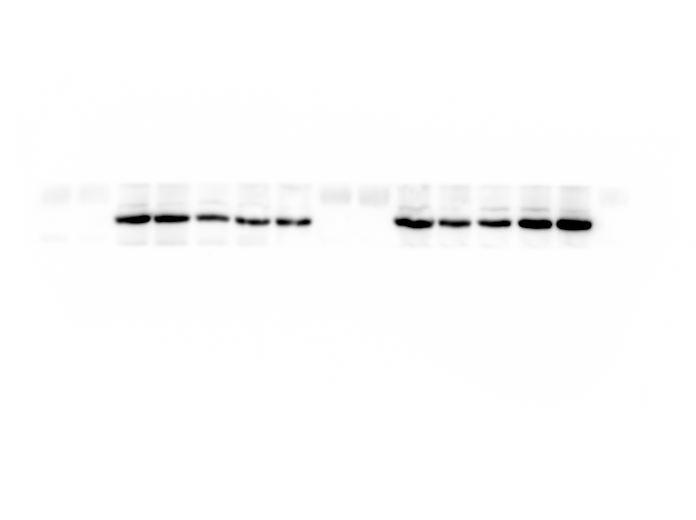


α-SMA 42kDa


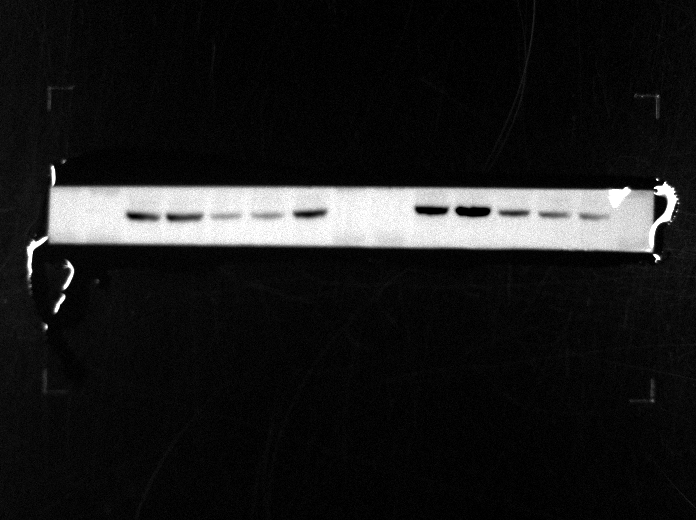


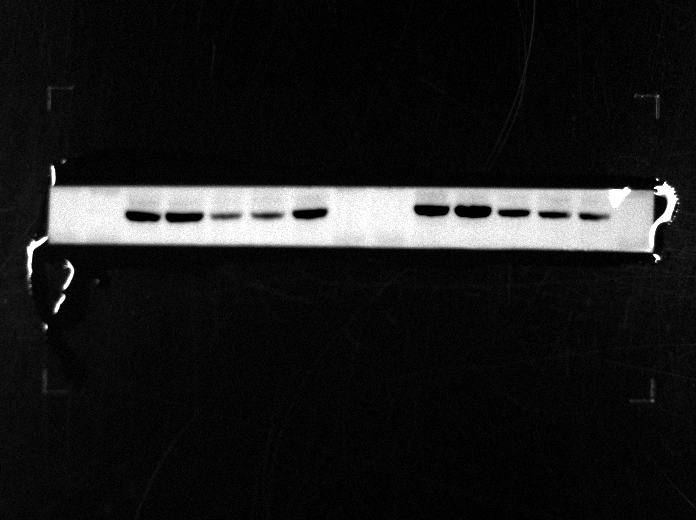


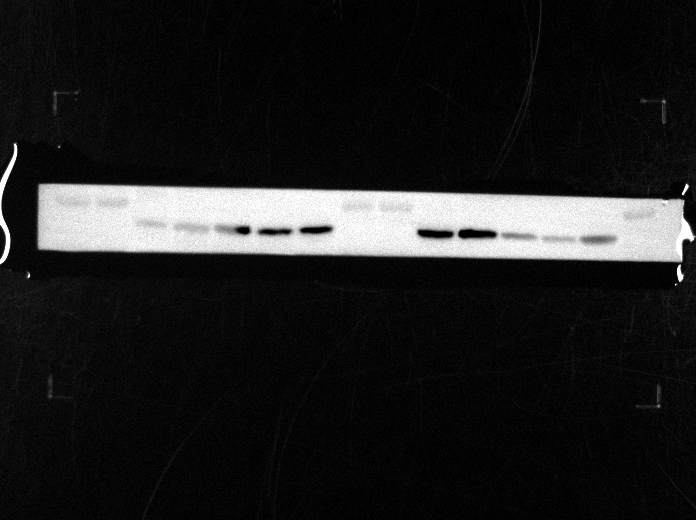


GAPDH 36kDa


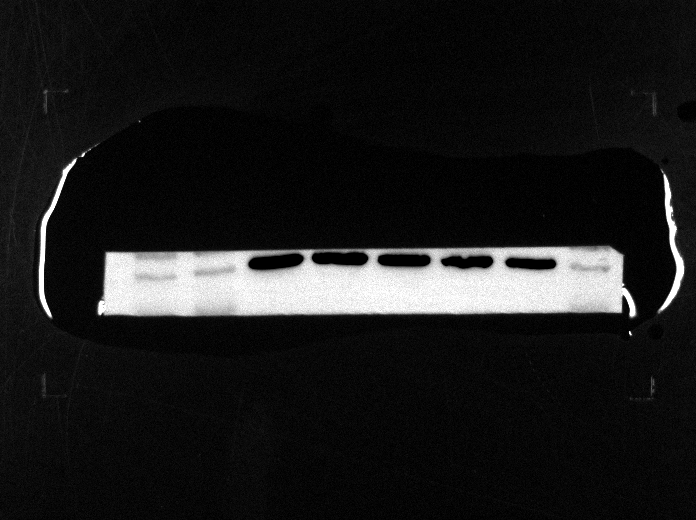


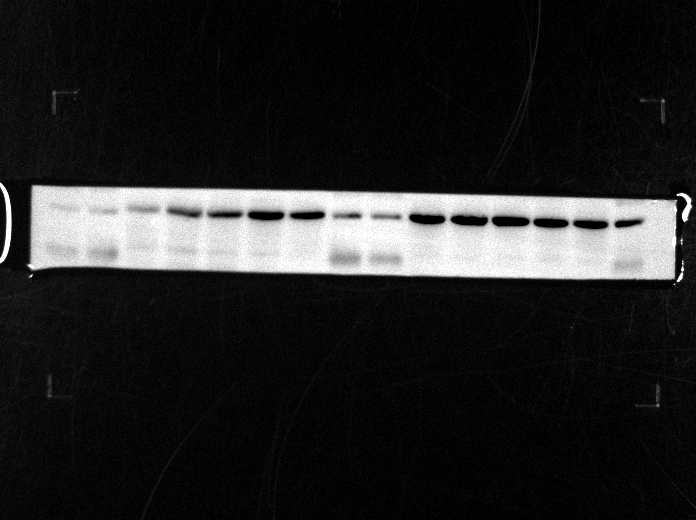


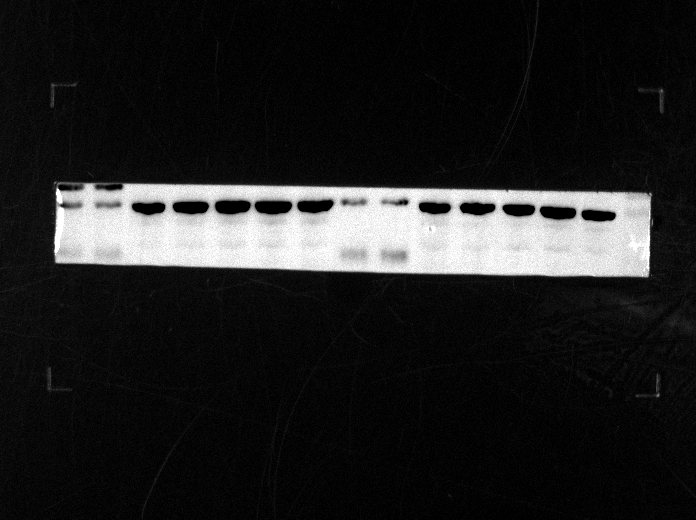


Supplementary file

Due to the overlap in the number of molecules in the strips and the fact that some of them could not be exposed together, we cropped them for exposure.
